# From Pan-Reactive K_V_7 Channel Opener to Subtype Selective Opener/Inhibitor by Addition of a Methyl Group

**DOI:** 10.1371/journal.pone.0100209

**Published:** 2014-06-23

**Authors:** Sigrid Marie Blom, Mario Rottländer, Jan Kehler, Christoffer Bundgaard, Nicole Schmitt, Henrik Sindal Jensen

**Affiliations:** 1 Division of Neuroscience Drug Discovery, H. Lundbeck A/S, Copenhagen, Denmark; 2 Division of Discovery Chemistry and DMPK, H. Lundbeck A/S, Copenhagen, Denmark; 3 Department of Biomedical Sciences and Danish National Research Foundation Centre for Cardiac Arrhythmia, Faculty of Health and Medical Sciences, University of Copenhagen, Copenhagen, Denmark; Sackler Medical School, Tel Aviv University, Israel

## Abstract

The voltage-gated potassium channels of the K_V_7 family (K_V_7.1–5) play important roles in controlling neuronal excitability and are therefore attractive targets for treatment of CNS disorders linked to hyperexcitability. One of the main challenges in developing K_V_7 channel active drugs has been to identify compounds capable of discriminating between the neuronally expressed subtypes (K_V_7.2–5), aiding the identification of the subunit composition of K_V_7 currents in various tissues, and possessing better therapeutic potential for particular indications. By taking advantage of the structure-activity relationship of acrylamide K_V_7 channel openers and the effects of these compounds on mutant K_V_7 channels, we have designed and synthesized a novel K_V_7 channel modulator with a unique profile. The compound, named SMB-1, is an inhibitor of K_V_7.2 and an activator of K_V_7.4. SMB-1 inhibits K_V_7.2 by reducing the current amplitude and increasing the time constant for the slow component of the activation kinetics. The activation of K_V_7.4 is seen as an increase in the current amplitude and a slowing of the deactivation kinetics. Experiments studying mutant channels with a compromised binding site for the K_V_7.2–5 opener retigabine indicate that SMB-1 binds within the same pocket as retigabine for both inhibition of K_V_7.2 and activation of K_V_7.4. SMB-1 may serve as a valuable tool for K_V_7 channel research and may be used as a template for further design of better subtype selective K_V_7 channel modulators. A compound with this profile could hold novel therapeutic potential such as the treatment of both positive and cognitive symptoms in schizophrenia.

## Introduction

The neuronally expressed members of the K_V_7 family (K_V_7.2–5) are the molecular correlates of the M-current, a slowly activating and slowly deactivating potassium current [1], [2], [3]. K_V_7 channels open at voltages below the threshold required for generation of an action potential and play a fundamental role in the control of neuronal excitability. Accordingly, mutations in the genes encoding K_V_7.2 and K_V_7.3 result in a form of neonatal epilepsy [4], [5], [6]. Hence, drugs that increase the activity of K_V_7 channels have a great therapeutic potential for the treatment of diseases characterized by hyperexcitability, such as epilepsy and neuropathic pain. Retigabine (Trobalt/Potiga), which activates K_V_7.2–5, was approved as an add-on treatment for partial onset seizures in 2011 and has proven effective in preclinical models for a wide variety of diseases [7], [8], [9], [10].

Based on mutation studies, retigabine has been shown to bind to a hydrophobic pocket between transmembrane segments 5 and 6 of the channel proteins. Specifically, channel activation by retigabine is critically dependent on a tryptophan residue (W236 in K_V_7.2) in the cytoplasmic part of S5 [11], [12]. The cardiac K_V_7.1 channel has a leucine at this position explaining its resistance to retigabine-induced enhancement. It appears that the tryptophan residue constitutes a structural element of a promiscuous binding site in the channels, since compounds which are structurally different from retigabine, like BMS-204352, (S)-1 and (S)-2 also lose their activating effects when the tryptophan is substituted for a leucine [13], [14]. Yet, compounds like ZnPy [15], ICA-27243 [16], [17] and fenamic acids [18] are not dependent on its integrity, suggesting that other activator binding sites exist. A leucine within the pore loop and a leucine extending from S6 of the adjacent subunit have also been found to be important residues for retigabine sensitivity [19]. These residues are conserved between the neuronal subtypes whereas K_V_7.1 carries valines in both positions. Introduction of the three critical residues in K_V_7.1 confers full retigabine sensitivity [19].

**Figure 1:**
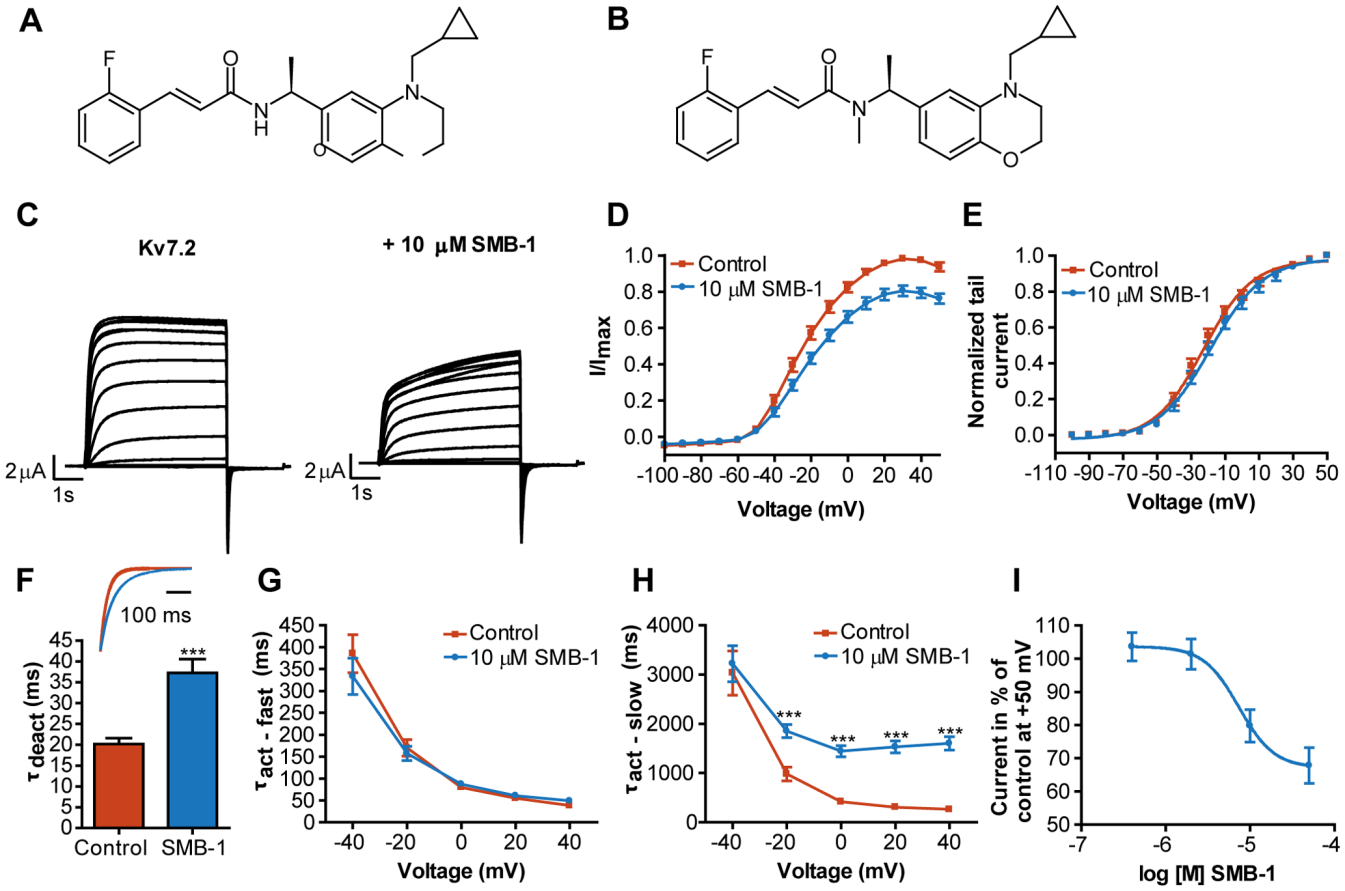
Inhibition of K_V_7.2 by SMB-1. Chemical structure of (S)-2 (**A**) and SMB-1 (**B**). (**C**) Representative current traces for K_V_7.2 in the absence and presence of 10 µM SMB-1 (**D**) Effect of SMB-1 on current-voltage relationship. (**E**) Effect of SMB-1 on voltage-dependence of activation. (**F**) Effect of SMB-1 on deactivation kinetics. Statistical significance was determined by paired, two-tailed Student's *t*-test. Representative tail current traces in the absence and presence of 10 µM SMB-1 are shown in the inset. Effect of SMB-1 on the fast (**G**) and slow (**H**) component of the activation kinetics. Statistical significance was determined by two-way repeated measurements ANOVA followed by Bonferroni post-test. Y-values were log-transformed before the statistical analysis to meet the assumption of normality. (**I**) Dose-response relationship for the effect of SMB-1 on K_V_7.2. *** *p<*0.001. Bars represent S.E.M and *n = *5–9. Note that the error bars in some instances are too small to be visible.

We have previously shown that the acrylamide (S)-2 (fig. 1A) activates K_V_7.2–5 [14]. For K_V_7.4 and K_V_7.5 the effect of (S)-2 is purely positive, while the compound has a bimodal effect on homomeric K_V_7.2 and heteromeric K_V_7.2/3 channels. For K_V_7.2, the positive effects of (S)-2 are a hyperpolarizing shift in the voltage-dependence of activation, a slowing of the deactivation kinetics (τ_deact_) and an acceleration of the fast component of the activation kinetics (τ_act_fast_). At voltages below −10 mV the compound also increases the current amplitude and accelerates the slow component of the activation kinetics (τ_act_slow_). However, at voltages above −10 mV the compound has a secondary inhibitory effect. At these depolarized voltages the effect on the current amplitude and τ_act_slow_ crosses over and becomes inhibitory. When we tested (S)-2 on a mutated K_V_7.2 channel where the tryptophan residue in S5 (which is essential for the effect of retigabine) was substituted for a leucine (K_V_7.2-W236L) all activating effects of (S)-2 were lost and the compound became purely inhibitory. Based on these observations we proposed that the mechanism behind the inhibitory effect on K_V_7.2-W236L and the secondary inhibitory action at the WT K_V_7.2 may be the same and that the effect of (S)-2 on K_V_7.2 can be divided into two entities: 1) an activating part visible as a shift in the voltage-dependence of activation and 2) an inhibitory part visible as a decrease in the current amplitude and an increase in τ_act_slow_. According to this proposal, the interpretation of the experimental data is that the W236L mutation disables only the (S)-2 induced activation and thereby unmasks the inhibitory component of the drug effect. Consistent with this proposal is the observation that for K_V_7.4, where the effect of (S)-2 is purely positive, the retigabine insensitive mutant (K_V_7.4-W242L) is fully insensitive to (S)-2.

This raised the question of whether it would be possible to design a compound that mimics the effect of the W-L mutation, resulting in an inhibitor of K_V_7.2 that would not affect K_V_7.4. Interestingly, it is described in the structure activity-relationship of the acrylamide class of compounds that methylation of the amide nitrogen of a related acrylamide K_V_7.2 opener (S)-1 results in a compound that inhibits K_V_7.2 [20]. In our hands (S)-1 is also an inhibitor of K_V_7.2-W236L (unpublished observation) and has been reported to be an inhibitor of K_V_7.4-W242L [13]. We hypothesized that this chemical modification of the acrylamide may have the same effect as the W-L mutation where the methylated version of (S)-1 would be an inhibitor of both K_V_7.2 and K_V_7.4, while the methylated version of (S)-2 would inhibit K_V_7.2 but not K_V_7.4. Here we describe the synthesis and characterization of an analogue of (S)-2, termed SMB-1, where the amide nitrogen is methylated (fig. 1B). We show that SMB-1 is indeed an inhibitor of K_V_7.2. In contrast, K_V_7.4 is activated by SMB-1. However, SMB-1 does not have sufficient penetration into the rodent brain to allow examination of its *in vivo* CNS profile.

## Materials and Methods

### Molecular biology

Point mutations were introduced using mutated oligonucleotide extension (PfuTurbo Polymerase, Stratagene, La Jolla, CA, USA) from a plasmid template harboring the cDNA of interest, digested with DpnI (Fermentas, St. Leon-Roth, Germany) and transformed into *E.coli* XL1 Blue cells. The construct was verified by complete DNA sequencing of the cDNA insert. cRNA was prepared from linearized human wild-type (WT) and mutant K_V_7 channels in the pGEM-HE vector using the T7 m-Message Machine kit (Ambion, Austin, TX, USA) according to the manufacturer's instructions.

### Electrophysiology

Extraction of *Xenopus laevis* oocytes and injection of cRNA was performed as described previously [14]. Oocytes were kept in Modified Barth's Saline (in mM: 88 NaCl, 1 KCl, 2.4 NaHCO_3_, 0.41 CaCl_2_, 0.82 MgSO_4_, 0.3 Ca(NO_3_)_2_, 15 HEPES, pH 7.4 supplemented with 100 U/mL penicillin and 100 µg/mL streptomycin) at 18°C, and currents were recorded after 2-7 days. The care of *Xenopus laevis* and the oocyte extraction procedure were performed according to national guidelines and approved by the Danish Animal Experiments Inspectorate.

K_V_7 currents in *Xenopus laevis* oocytes were recorded using two-electrode voltage-clamp at room temperature in Ringer buffer (in mM: 115 NaCl, 2.5 KCl, 1.8 CaCl_2_, 0.1 MgCl_2_, 10 HEPES, pH 7.4) as described previously [14]. Data was acquired using pCLAMP 10.2 software (Molecular Devices, CA, USA) and analyzed using pCLAMP 10.2 and GraphPad Prism 4.0 (GraphPad Software Inc., CA, USA). Currents were elicited from a holding potential of −80 mV by 5 s steps to potentials between −100 and +50 mV in 10 mV increments, followed by a 2 s step to −120 mV.

SMB-1 was dissolved in dimethyl sulfoxide (DMSO) to obtain a concentrated stock solution. On the day of experiments the stock solution was thawed and diluted in Ringer buffer to the final concentrations. A concentration of 10 µM SMB-1 was used unless indicated otherwise. The final DMSO concentration never exceeded 0.1%.

### Curve fitting and statistical analysis

Current – voltage (*I*–V) relationship curves were generated by normalizing the steady-state peak current measured at potentials between −100 and +50 mV to the current measured at +50 mV in control recordings and plotting the values against the test potential.

The voltage-dependence of activation was determined from tail current analysis using the current measured immediately after the transient capacitive current after stepping to −120 mV from potentials between −100 mV and +50 mV. Data was normalized to extend from 0–1 and the tail current-voltage relationship was fitted to the Boltzmann equation:
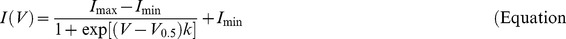
(Equation\;1)where *I*_max_ is the maximum tail current, *I*_min_ is the minimum tail current, V_0.5_ is the potential for half maximal activation and *k* is the slope factor. A V_0.5_ was calculated for each individual experiment and statistical significance was estimated by paired two-tailed Student's *t*-test.

Activation kinetics for K_V_7.2 and K_V_7.2-W236L was determined by fitting the traces recorded at potentials between −40 and +40 mV to a double exponential function: 
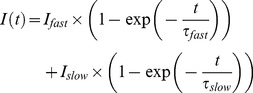
(Equation\;2)where *I(t)* is the current at time *t*, *I_fast_* and *I_slow_* are the current amplitudes at infinite times, and *τ_fast_* and *τ_slow_* are the time constants of the fast and slow components, respectively.

Activation kinetics for K_V_7.4 and K_V_7.4-W242L was determined by fitting the traces recorded at potentials between −30 and +50 mV to a single exponential function:
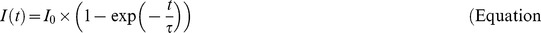
(Equation\;3)where *I(t)* is the current at time *t*, *I*_0_ is the peak current and τ is the activation time constant.

For investigation of deactivation kinetics the tail current traces recorded at −120 mV when stepping from the channels' respective ∼V_0.5_ were fitted to a single exponential function (Eq. 3).

Dose-response curves from electrophysiological experiments were made by plotting the increase in steady state current at +50 mV expressed in percentages as a function of drug concentration. The data were then analyzed by non-linear regression and fitted to the equation for sigmoidal dose-response with variable slope:
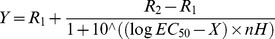
(Equation\;4)where *R_1_* is the initial response, *R_2_* is the maximum response, *X* is the logarithm of the drug concentration and *nH* is the slope (Hill coefficient) of the curve.

For data concerning activation kinetics statistical significance was determined by two-way ANOVA followed by Bonferroni post-tests (if the overall P-value of the drug factor was less than 0.05). Before the statistical analyses of the fast component of the activation for K_V_7.2 and K_V_7.2-W236L the measured time constants were log-transformed to meet the assumption of normality. For traces fitted to double exponential functions only those where the sum of *I_fast_* and *I_slow_* equaled the peak current were included in the analysis.

For analysis of the remaining data statistical significance was determined by two-tailed Student's *t*-test if single comparisons were made and by two-way ANOVA followed by Bonferroni post-tests if multiple comparisons were made. Statistical analyses were carried out using GraphPad Prism 4.0. *p<*0.05 was accepted for statistical difference. All values are shown as mean ± S.E.M.

### Rodent exposure studies

Ethical permission for the *in vivo* rat procedures used in these studies was granted by the animal welfare committee, appointed by the Danish Ministry of Justice and all animal procedures were carried out in compliance with EC Directive 86/609/EEC and with the Danish law regulating experiments on animals.

Brain and plasma exposure of SMB-1 was evaluated in male Sprague-Dawley rats (225–250 g, Charles River Lab., UK). SMB-1 was administered subcutaneously in solution at a dose of 20 mg/kg in 25% solutol (dose volume 5 ml/kg) and exposure was assessed after 30 and 120 min (n = 3). Under isoflurane anesthesia, cardiac blood was obtained in EDTA-coated tubes and centrifuged for 10 min at 4°C, after which plasma was harvested. While still under anesthesia, the animal was decapitated, the brain was removed and brain homogenate was prepared by homogenizing the whole brain with 70% acetonitrile (1∶4 v/v) followed by centrifugation and collection of the supernatant. Concentrations of SMB-1 were determined using turboflow chromatography (dual column, focus mode; Thermo Fisher Scientific, Waltham, MA) followed by tandem mass spectrometry detection (Sciex API-3000 MS; Applied Biosystems, Foster City, CA). The limit of detection was 1 ng/ml in plasma and 5 ng/g in brain.

### Membrane permeability and free fraction determination

The bidirectional permeability (P_app_) of SMB-1 was examined in an MDCK cell system expressing human MDR1 [ABCB1, P-glycoprotein (P-gp)] as described previously [21]. Permeation of the test compound from apical (A) to basal (B) direction or B to A direction was determined in triplicate over a 150-min incubation at 37°C. The efflux ratio was calculated as the ratio between P_app_ (B to A) and P_app_ (A to B). Free fraction of SMB-1 was determined using a standard equilibrium dialysis methodology with freshly isolated rat brain homogenate or plasma [22]. Equilibrium dialysis was performed by incubating at 37°C for 5 h in triplicates.

### Chemistry

(S)-2 was prepared according to a literature procedure [23]. Synthesis of SMB-1 ((E)-N-[(S)-1-(4-Cyclopropylmethyl-3,4-dihydro-2H-1,4-benzoxazin-6-yl)-ethyl]-3-(2-fluoro-phenyl)-N-methyl-acrylamide): To a solution of (S)-2 (100 mg, 262.8 µmol) in a mixture of anhydrous tetrahydrofuran (THF, 1.5 mL) and dimethylformamide (DMF, 0.3 mL) at 0°C was added NaH (16 mg, 394 µmol, 60% dispersion in mineral oil) and MeI (17.8 µL, 289 µmol). The mixture was stirred at 0°C for four hours and at room temperature overnight. The mixture was cooled to 0°C, and diluted with ethylacetate (EtOAc, 10 mL). Water (10 mL) was added carefully, the organic phase was separated, and the aqueous layer was extracted by EtOAc. The combined organic layers were washed by brine, dried (NaSO_4_), and concentrated to give SMB-1 (104 mg, yield: 100%) as an oil. ^1^H NMR (CDCl_3_ 400 MHz TMS): δ7.63–7.53 (m, 1 H), 7.35–7.25 (m, 1 H), 7.15–7.05 (m, 1 H), 7.01–6.78 (m, 3 H), 6.58–6.30 (m, 3 H), 5.90–5.82 (m, 0.58 H), 5.12–5.02 (m, 0.33 H), 4.06 (t, J = 4.4 Hz, 2 H), 3.22 (t, J = 4.4 Hz, 2 H), 2.95–2.86 (m, 2 H), 2.61 (s, 3 H), 1.45–1.36 (m, 1 H), 1.28 (d, J = 6.8 Hz, 2 H), 0.84–0.74 (m, 1 H), 0.38–0.30 (m, 2 H), 0.50–0.00 (m, 2 H).

## Results

### SMB-1 is an inhibitor of K_V_7.2

We heterologously expressed K_V_7 channels in *Xenopus laevis* oocytes and assessed their biophysical properties by two-electrode voltage-clamp. Upon activating voltage-steps, K_V_7.2 channels displayed slowly activating and deactivating currents as expected [1]. Representative current traces are shown in Figure 1C. Application of 10 µM SMB-1 led to a reduction of current amplitudes at all activating potentials (fig. 1C and D). At +50 mV the current amplitude was 81.7±3.1% (*n = *9) of control. A small, but significant, shift to more positive potentials was observed in the voltage-dependence of activation (V_0.5_: control: −22.5±2.2 mV; SMB-1: -18.0±2.7 mV, *n = *8, *p<*0.05, fig. 1E). We also assessed kinetic parameters by determining the time constants of deactivation (τ_deact_) and activation (τ_act_fast_ and τ_act_slow_). Deactivation kinetics were significantly slowed in the presence of SMB-1 (control: 20.2±1.4 ms, SMB-1: 37.3±3.3 ms, *n = *7, *p<*0.001, fig. 1F). SMB-1 did not affect τ_act_fast_ (fig. 1G) but increased τ_act_slow_ at −20 mV and above (fig. 1H). These effects of SMB-1 are remarkably similar to the effect of (S)-2 on K_V_7.2-W236L [14].

To determine the potency of SMB-1 on K_V_7.2 the compound was tested at four concentrations (0.4, 2, 10 and 50 µM). Visual inspection of the solution indicated that the drug precipitated at concentrations above 50 µM. The current amplitude in percentage of control at +50 mV was plotted against the logarithm of the concentration. Fitting the data to the equation for sigmoidal dose-response with variable slope determined the IC_50_ value of SMB-1 to ∼7.4 µM (pIC_50_: 5.1±0.2 M, *n = *5, fig. 1I). The compound was not able to fully inhibit the current within the concentration range tested, reaching a maximal inhibition of 67.6±5.3% of control (*n = *5) at +50 mV.

### SMB-1 is an activator of K_V_7.4

**Figure 2:**
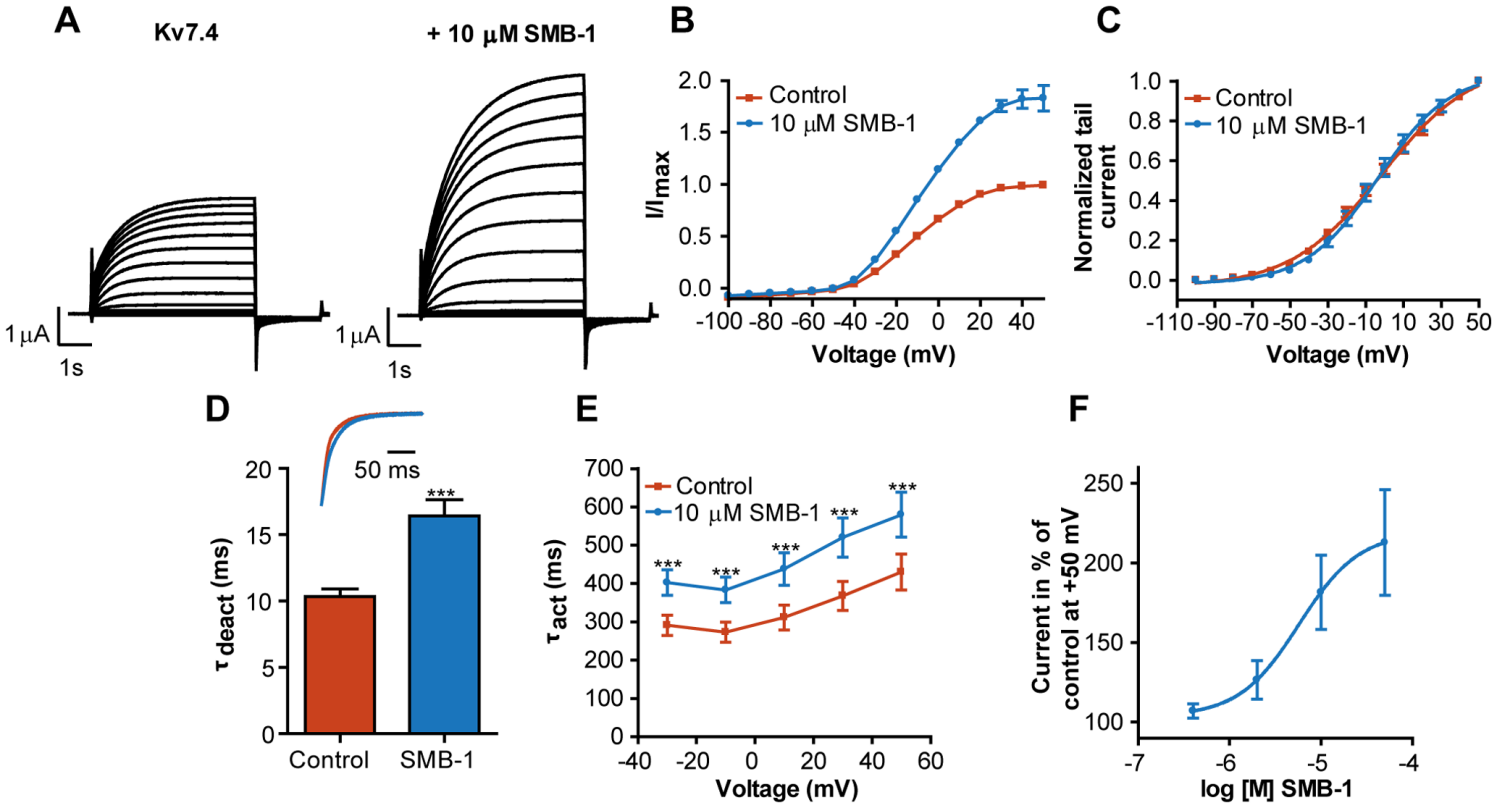
Activation of K_V_7.4 by SMB-1. (**A**) Representative current traces for K_V_7.4 in the absence and presence of 10 µM SMB-1. (**B**) Effect of SMB-1 on current-voltage relationship. (**C**) Effect of SMB-1 on voltage-dependence of activation. (**D**) Effect of SMB-1 on deactivation kinetics. Statistical significance was determined by paired, two-tailed Student's *t*-test. Representative tail current traces in the absence and presence of 10 µM SMB-1 are shown in the inset. (**E**) Effect of SMB-1 on activation kinetics. Statistical significance was determined by two-way repeated measurements ANOVA followed by Bonferroni post-test. (**F**) Dose-response relationship of SMB-1 on K_V_7.4. *** *p<*0.001. Bars represent S.E.M and *n = *5–8.

Next, we addressed the effect of SMB-1 on K_V_7.4. Submitting *Xenopus laevis* oocytes expressing K_V_7.4 channels to a voltage-step protocol elicited slowly activating currents. A representative experiment is shown in Figure 2A. Application of SMB-1 resulted in increased current amplitudes at −40 mV and above (fig. 2A and B). The current amplitude was 184.5±12.4% of control (*n = *8) at +50 mV in the presence of 10 µM SMB-1, which is low compared to the efficacy of (S)-2 on K_V_7.4 (1927% of control at +40 mV) [14]. SMB-1 did not affect the voltage-dependence of activation of K_V_7.4 (fig. 2C, V_0.5_: control: −0.2±3.6 mV; 10 µM SMB-1: −1.4±3.4 mV, *n = *8, *p = *0.59). Yet, 10 µM SMB-1 significantly increased τ_deact_ (control: 10.3±0.6 ms; SMB-1: 16.4±1.2 ms, *n = *8, *p<*0.001, fig. 2D), and τ_act_ at all potentials tested (fig. 2E). The EC_50_ value was determined to 5.7 µM (pEC_50_: 5.2±0.4 M, *n = *5) and the maximal efficacy to 212.7±33.1% (*n = *5) at +50 mV (fig. 2F).

### Dependency on the critical tryptophan residue for inhibition/activation by SMB-1

**Figure 3:**
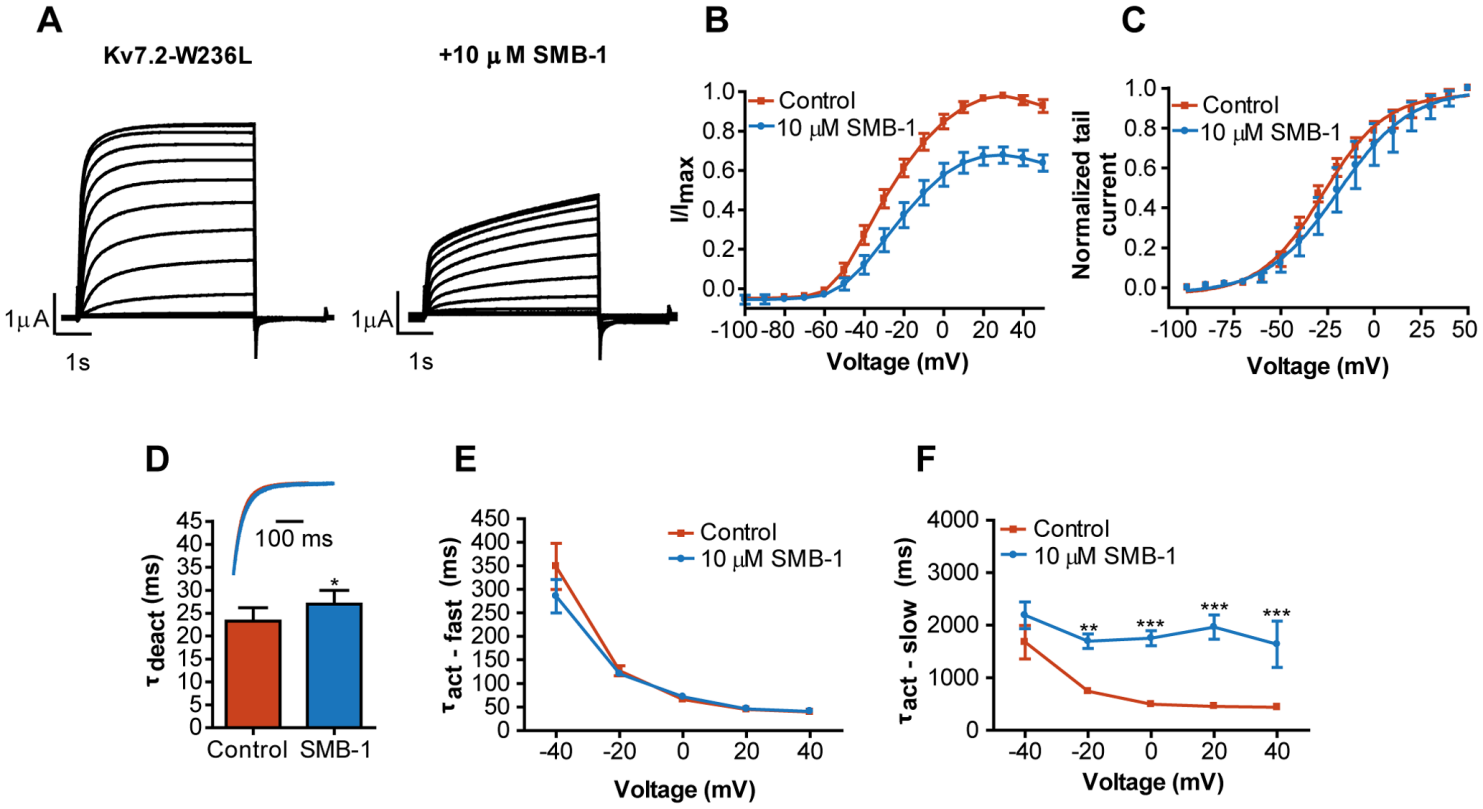
Effect of SMB-1 on K_V_7.2-W236L. (**A**) Representative current traces for K_V_7.2-W236L in the absence and presence of 10 µM SMB-1 (**B**) Effect of SMB-1 on current-voltage relationship. (**C**) Effect of SMB-1 on voltage-dependence of activation. (**D**) Effect of SMB-1 on deactivation kinetics. Statistical significance was determined by paired, two-tailed Student's *t*-test. Representative tail current traces in the absence and presence of 10 µM SMB-1 are shown in the inset. Effect of SMB-1 on the fast (**E**) and slow (**F**) component of the activation kinetics. Statistical significance was determined by two-way repeated measurements ANOVA followed by Bonferroni post-test. Y-values were log-transformed before the statistical analysis to meet the assumption of normality. * *p<*0.05, ** *p<*0.01, *** *p<*0.001. Bars represent S.E.M and *n = *4–6.

**Figure 4:**
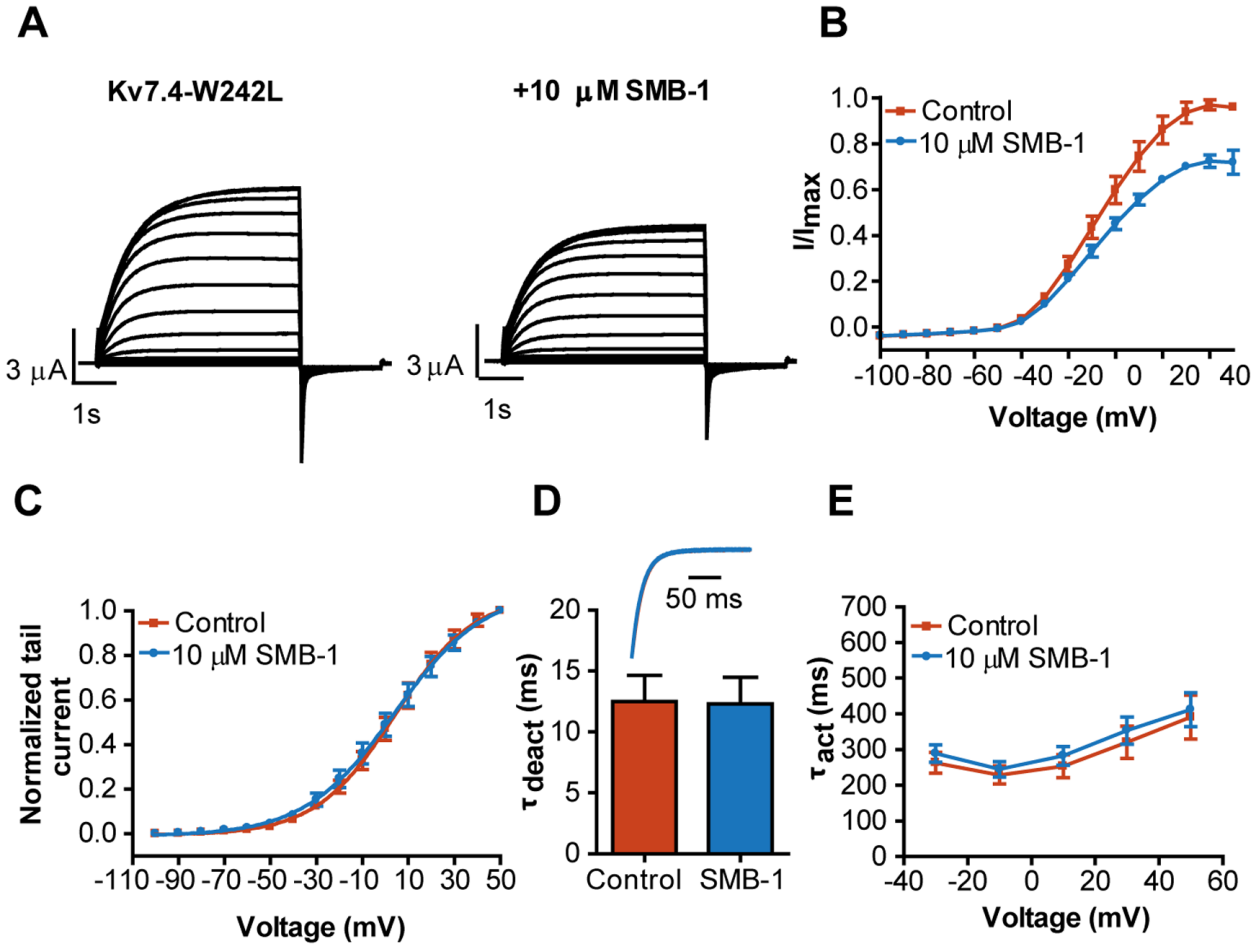
Effect of SMB-1 on K_V_7.4-W242L. (**A**) Representative current traces for K_V_7.4-W242L in the absence and presence of 10 µM SMB-1. (**B**) Effect of SMB-1 on current-voltage relationship. (**C**) Effect of SMB-1 on voltage-dependence of activation. (**D**) Effect of SMB-1 on deactivation kinetics. Statistical significance was determined by paired, two-tailed Student's *t*-test. Representative tail current traces in the absence and presence of 10 µM SMB-1 are shown in the inset (note that the traces are completely overlapping). (**E**) Effect of SMB-1 on activation kinetics. Statistical significance was determined by two-way repeated measurements ANOVA followed by Bonferroni post-test. Bars represent S.E.M and *n = *5.

To determine whether SMB-1 was dependent on the critical tryptophan residue in the ‘retigabine binding-site' for its inhibitory/activating effects, the compound was applied to mutant channel proteins K_V_7.2-W236L and K_V_7.4-W242L (fig. 3 and 4). K_V_7.2-W236L was inhibited by SMB-1 similar to wild-type (WT) K_V_7.2 (fig. 3A and B). The current amplitude at +50 mV was 68.8±3.9% of control (*n = *6). We observed a trend towards an increase in the half-maximal value for voltage-dependence of activation V_0.5_ (control: −27.1±2.4 mV; SMB-1: −15.0±10.1 mV, *n* = 4, *p = *0.2, fig. 3C). There was a small but significant increase in τ_deact_ (control: 23.3±2.9 ms; SMB-1: 27.0±3.0 ms, *n* = 6, *p<*0.05, fig. 3D). Like for WT K_V_7.2, there was no significant effect of SMB-1 on τ_act_fast_ (fig. 4E), while τ_act_slow_ was significantly increased at potentials above −20 mV (fig. 3F).

In contrast, the activating effect of SMB-1 on K_V_7.4 was abolished in the W242L mutant channel; the mutant channel was inhibited by the compound (fig. 4A and B). The current amplitude was 74.8±4.2% of control (*n* = 5) at +50 mV. At 10 µM, SMB-1 did not affect the voltage-dependence of activation (control: V_0.5_ = 5.5±4.5 mV; SMB-1: V_0.5_ = 4.3±4.9 mV, *n* = 5, *p = *0.2, fig. 4C) or the deactivation kinetics of K_V_7.4-W242L (control: τ_deact_ = 12.5±2.2 ms; SMB-1: τ_deact_ = 12.3±2.2 ms, *n* = 5, *p = *0.5, fig. 4D), and there was neither any significant effect on activation kinetics (fig. 4E).

Hence, the activating effect of SMB-1 on K_V_7.4 is critically dependent on the tryptophan residue while the inhibitory effect on K_V_7.2 is not.

### Effect of SMB-1 on channels with mutations in the refined retigabine binding site

**Figure 5:**
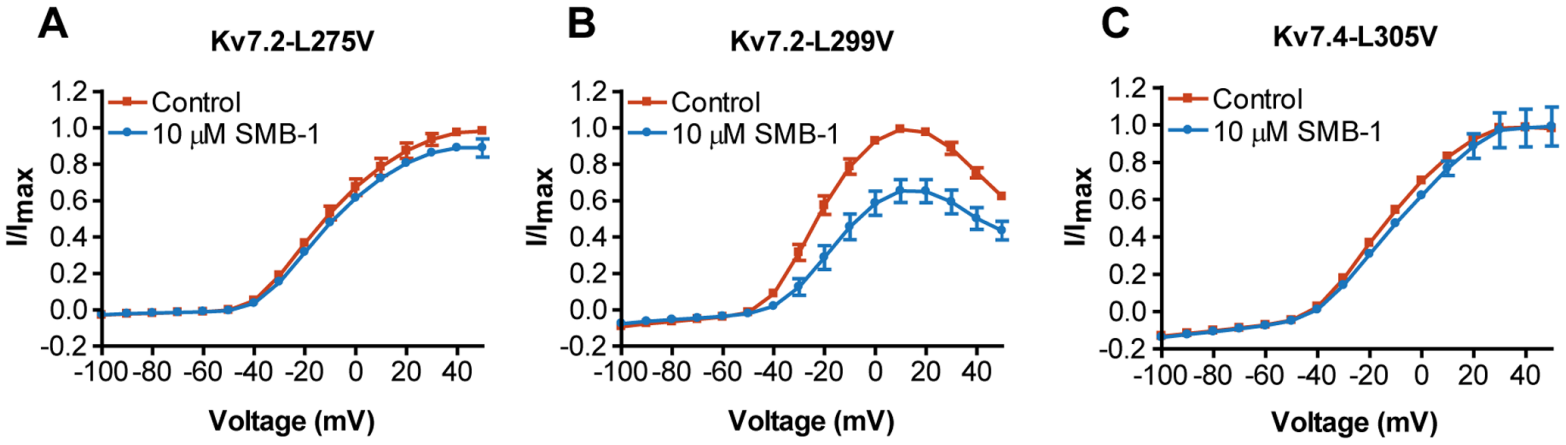
Effect of SMB-1 on channels with mutations in the refined retigabine binding site. Effect of 10 µM SMB-1 on the current-voltage relationship of (A) K_V_7.2-L275V, (B) K_V_7.2-L299V and (C) K_V_7.4-L305V. Bars represent S.E.M. and *n = *4–6.

To test if SMB-1 depends on other residues important for the action of retigabine, we generated channels where the two leucine residues (L275 and L299 in K_V_7.2, L281 and L305 in K_V_7.4) shown to be part of the binding site in K_V_7.3 [19] were substituted with valines, the corresponding residues in K_V_7.1. The resulting channels K_V_7.2-L275V, K_V_7.2-L299V, K_V_7.4-L281V and K_V_7.4-L305V were expressed in *Xenopus laevis* oocytes. We determined their *I*-V relationship in the absence and presence of 10 µM SMB-1 (fig. 5). All channels behaved similar to the WT channels but displayed a voltage-dependence of activation that was shifted in the positive (K_V_7.2-L275V: V_0.5_ = −7.7±1.1 mV, *n* = 36 and K_V_7.2-L299V: V_0.5_ = −8.7±1.3 mV, *n* = 30) or negative (K_V_7.4-L305V: V_0.5_ = −10.7±2.2 mV, *n* = 37) direction. The L281V mutation in K_V_7.4 resulted in a non-functional channel. A mutation affecting this residue (L281S) has been found in patients with DFNA2 [24], indicating that it may be very critical for K_V_7.4 channel function.

The L275V mutation in K_V_7.2 affected the efficacy of SMB-1 (fig. 5A). The current amplitude was 90.4±4.1% of control at +50 mV (*n* = 4). K_V_7.2-L299V was inhibited to a similar degree as the WT channel (current amplitude 69.3±5.6% of control at +50 mV, *n* = 4, fig. 5B). The L305V mutation in K_V_7.4 abolished the effect of the compound (current amplitude 100.8±10.3% of control at +50 mV, *n* = 6, fig. 5C).

### Exposure of SMB-1 in rats

Following subcutaneous administration of 20 mg/kg, SMB-1 was found to be rapidly absorbed systemically, and high plasma concentrations were observed (Table 1). In contrast, we found the brain exposure to be very low relative to plasma with corresponding brain/plasma partition coefficients in the range of 0.04–0.07. Considering the contribution of residual blood in the brain tissue to the brain partition coefficient, these results suggest that SMB-1 possesses limited brain penetration capabilities. In line with these findings, *in vitro* transport studies in MDCK cells showed that SMB-1 exhibited low membrane permeability of 2.5±0.3×10^−6^ cm/s with an efflux ratio of 0.7. In addition, SMB-1 was found to be highly bound in plasma (free fraction 0.6±0.4%) and in brain homogenate (free fraction 0.1±0.1%) (mean ± S.E.M., n = 3).

**Table 1:** Plasma and brain exposure of SMB-1 in rats following subcutaneous administration of 20 mg/kg.

Time (min)	Plasma (µM)	Brain (µM)
30	14±1.5	0.55±0.04
120	6.4±0.70	0.44±0.07

Data shown as mean total concentrations ± S.E.M (n = 3).

## Discussion

Although an increasing number of compounds has been found to activate or inhibit K_V_7 channels there is still a lack of truly subtype specific drugs that can discriminate between K_V_7.2–5. This has hampered the determination of the individual contribution of each subtype to the *in vivo* effects of e.g. retigabine and made it difficult to establish which subtype to target for each of the many indications where K_V_7 channels have been suggested as attractive therapeutic targets.

In an attempt to develop subtype-specific K_V_7 active drugs, we took advantage of previous findings that had established the so-called retigabine binding site and the SAR of acrylamide K_V_7 channel compounds [11], [12], [19], [20]. Trying to mimic the effect of a tryptophan substitution, we designed and synthesized a methylated version of (S)-2, termed SMB-1. This compound oppositely affects K_V_7.2 and K_V_7.4 channels.

SMB-1 inhibits K_V_7.2 by reducing the current amplitude and increasing the time constant for the slow component of the activation kinetics remarkably similar to the effects of (S)-2 on K_V_7.2-W236L [14]. The potency of (S)-2 on K_V_7.2-W236L (IC_50_ = 8.1 µM) and SMB-1 on K_V_7.2 (IC_50_ = 7.4 µM) is similar [14]. Hence, our data suggests that methylation of the amide nitrogen disables the activating component of (S)-2's effect on K_V_7.2 while preserving the inhibitory component, and thereby recapitulates the pharmacological consequence of the W236L mutation.

The increase in K_V_7.4 current levels by SMB-1 was weak compared to the high efficacy of (S)-2 [14]. As K_V_7.4-W242L was inhibited by SMB-1, we speculate that the methyl-group in SMB-1 promotes an inhibitory binding mode and thereby tips the activation/inhibition equilibrium towards reduced efficacy relative to the effects of (S)-2 on K_V_7.4. The positive effect of SMB-1 on K_V_7.4 was critically dependent on the tryptophan residue in S5. Furthermore, K_V_7.4-L305V was SMB-1 insensitive, leading us to conclude that SMB-1 binds to the promiscuous retigabine binding site for activation of K_V_7.4.

In contrast, the inhibitory action of SMB-1 on K_V_7.2 was not critically dependent on the tryptophan residue. However, the L275V mutation in K_V_7.2 reduced the inhibitory efficacy of 10 µM SMB-1, indicating that it's binding to K_V_7.2 also occurs within the border of this binding pocket. Since this site accommodates pan-reactive compounds, it has been suggested that it would not allow for discrimination between neuronal K_V_7 channel subtypes [25]. These findings demonstrate that this binding site can indeed accommodate compounds that oppositely affect neuronal K_V_7 channels thereby giving rise to subtype selectivity. To further examine the basis of the opposite effect of SMB-1 on K_V_7.2 and K_V_7.4 in more detail, a cross-mutational approach may be applied where non-conserved residues within the binding pocket are swapped between the two subtypes.

What may this compound be used for? Foremost, determining the contribution of different K_V_7 channel subtypes to currents in various regions may assign particular physiological functions to particular subtypes.

Compounds modulating K_V_7 channels may have a therapeutic potential in relation to schizophrenia. This disease is characterized by psychotic or positive symptoms such as hallucinations, delusions, thought disorder, disorganized speech and paranoia; negative symptoms, which include lack of mental activities such as thoughts and motivation; and cognitive symptoms comprising failure of working memory, impairments of learning and attentional dysfunction [26]. According to the *dopamine hypothesis of schizophrenia* the positive symptoms result from over-active neurotransmission in the mesolimbic dopamine pathway [27], [28]. This hypothesis is based on the fact that antipsychotic drugs work by blocking dopamine D_2_ receptors, while compounds that increase dopamine levels can induce psychosis or exacerbate schizophrenia [27], [29]. On the other hand, a hypodopaminergic state in the frontal cortical terminal fields of the mesocortical dopamine neurons is thought to underlie the negative and cognitive symptoms [28]. The antipsychotic drugs available on the market today are more or less efficient in ameliorating the positive symptoms but have little or no effect on the negative and cognitive symptoms. Compounds with differential modulatory action across the K_V_7 channel family could aid in determining the most optimal subtype profile.

K_V_7 channel inhibitors were initially developed as cognition enhancers based on their ability to increase acetylcholine release [30], [31], [32]. This effect probably stems from inhibition of K_V_7.2 and K_V_7.2/3 channels as the reference inhibitor XE-991 has been shown to more potently inhibit K_V_7.2 and K_V_7.2/3 than K_V_7.4 channels albeit the data are reported from different laboratories [1], [33]. K_V_7 channels also play a role in the dopaminergic system and retigabine has anti-dopaminergic effects in the basal ganglia [9], [34], [35], [36]. K_V_7.4 has been suggested as the subtype mediating the efficacy of pan-reactive K_V_7 channel openers in modulation of the dopaminergic system [9], [35], [36]. Hence, we speculate that a balanced inhibition of K_V_7.2 and K_V_7.2/3 in combination with enhancement of K_V_7.4 current may be efficacious with respect to targeting of both positive and cognitive symptoms in patients suffering from schizophrenia. As this is hypothetical, proof of this concept necessitates an evaluation of SMB-1 in animal models of psychosis and cognition. Unfortunately, even though SMB-1 was not identified as a substrate for the efflux transporter P-gp judged from the efflux ratio in MDR1-MDCK cells, we found it to have low membrane permeability with poor CNS distribution in rats limiting its *in vivo* pharmacological potential after systemic administration in the CNS. However, the recent years have provided evidence for a role of the so-called neuronal K_V_7 channels in the vasculature [37], [38], [39]. K_V_7.4 channels in particular have been involved in the regulation of vascular tone in e.g. renal, coronary, and cerebral arteries [40], [41], [42]. Hence, further studies are warranted to investigate whether SMB-1 might have vasorelaxant effects which could in turn have an impact on cerebral circulation.

In conclusion, we have identified a novel K_V_7 channel active compound with a unique characteristic in that it inhibits K_V_7.2 but activates K_V_7.4. SMB-1 provides valuable insight into the pharmacology of K_V_7 channels and may be used as a template for the further design of better subtype selective K_V_7 channel modulators.
